# Ultra-pure platelet isolation from canine whole blood

**DOI:** 10.1186/1746-6148-9-144

**Published:** 2013-07-17

**Authors:** Shauna A Trichler, Sandra C Bulla, John Thomason, Kari V Lunsford, Camilo Bulla

**Affiliations:** 1 Department of Pathobiology and Population Medicine, College of Veterinary Medicine, Mississippi State University, Starkville, MS, USA; 2 Department of Clinical Sciences and Animal Health Center, College of Veterinary Medicine, Mississippi State University, Starkville, MS, USA

**Keywords:** Blood, Dog, Leukocyte contamination, Platelet, Purity

## Abstract

**Background:**

Several research applications involving platelets, such as proteomic and transcriptomic analysis, require samples with very low numbers of contaminating leukocytes, which have considerably higher RNA and protein content than platelets. We sought to develop a platelet purification protocol that would minimize contamination, involve minimal centrifugation steps, and yield highly pure platelet samples derived from low volume whole blood samples from healthy dogs.

**Results:**

Using an optimized OptiPrep density gradient technique, platelet recovery was 51.56% with 99.99% platelet purity and leukocyte contamination of 100 leukocytes per 10^8^ platelets, on average. Platelet samples were subjected to additional purification with CD45-labeled Dynabeads after density barrier centrifugation resulting in a 95-fold depletion of residual leukocytes. Platelets purified using these methods remained inactivated as assessed by Annexin V and P-selectin labeling with flow cytometry.

**Conclusions:**

The use of OptiPrep density gradient is a quick method for obtaining highly purified platelet samples from low volumes of canine whole blood with minimal contamination. Additional depletion of residual leukocytes can be achieved using CD45-labeled beads. These platelet samples can then be used for many downstream applications that require ultra-pure platelet samples such as RNA and protein analysis.

## Background

Platelets are known to play important roles in hemostasis and tissue repair. Recently, the role of platelets in angiogenesis is being evaluated more closely as a potential modulator of tumor progression and metastasis. However, the continued exploration of platelets and their roles in hemostasis, healing, or cancer progression and metastasis requires increasingly pure platelet isolates.

Newer methods being employed in the study of disease include proteomic, transcriptomic and metabolic studies that require platelet samples with very low levels of leukocyte contamination. Leukocytes have been estimated to contain 12,500-fold more mRNA and 65-fold more protein than platelets [1,2]. Therefore, even very low numbers of contaminating cells will produce significant contamination. Current commonly used methods for producing platelet rich plasma (PRP) can yield up to 72% platelet recovery but, in exchange for the high platelet yield, these samples often have high residual leukocyte counts (> 3,000 leukocytes per 10^8^ platelet) [3,4]. High levels of leukocyte contamination would produce a sample in which the majority of isolated mRNA is likely to be of leukocyte, rather than platelet, origin.

Methodologies that have successfully been used to produce highly pure platelet isolates devoid of notable leukocyte contamination require relatively large volumes of whole blood (40 mL or greater) to recover sufficient platelets for study as a significant percentage of the platelets are lost during the purification process [5]. High platelet purities may also be achieved by use of platelet aphaeresis during blood collection as well as use of specialized leukocyte filters. Specialized equipment and training are required for platelet apheresis collection methods, which are not widely available in the clinical veterinary practice setting [3,6]. Again, employing multiple filters for leukocyte depletion from platelet samples leads to considerable platelet loss, often requiring large volumes of whole blood or platelet concentrate [7]. The larger blood volumes required for these techniques also makes them impractical for use in preparing clinical veterinary samples for analysis. Other techniques for producing highly pure platelet samples often require multiple centrifugation steps. The physical stress of these centrifugation steps often leads to platelet activation and subsequent granule content release which alters results if looking at platelet contents via protein or RNA.

The dog has quickly gained recognition as an important animal model for many human diseases including cancer. Not only are dogs of veterinary importance from a companion animal standpoint, but also develop many of the same naturally occurring cancers and diseases as humans [8-10].Our goal was to provide a fast and reliable method for producing ultra-pure platelet samples suitable for proteomic and transcriptomic analysis from clinically relevant (3–5 mL) whole blood volumes with minimal platelet activation.

## Results

### Decreased leukocyte contamination using OptiPrep density barrier

**Figure 1:**
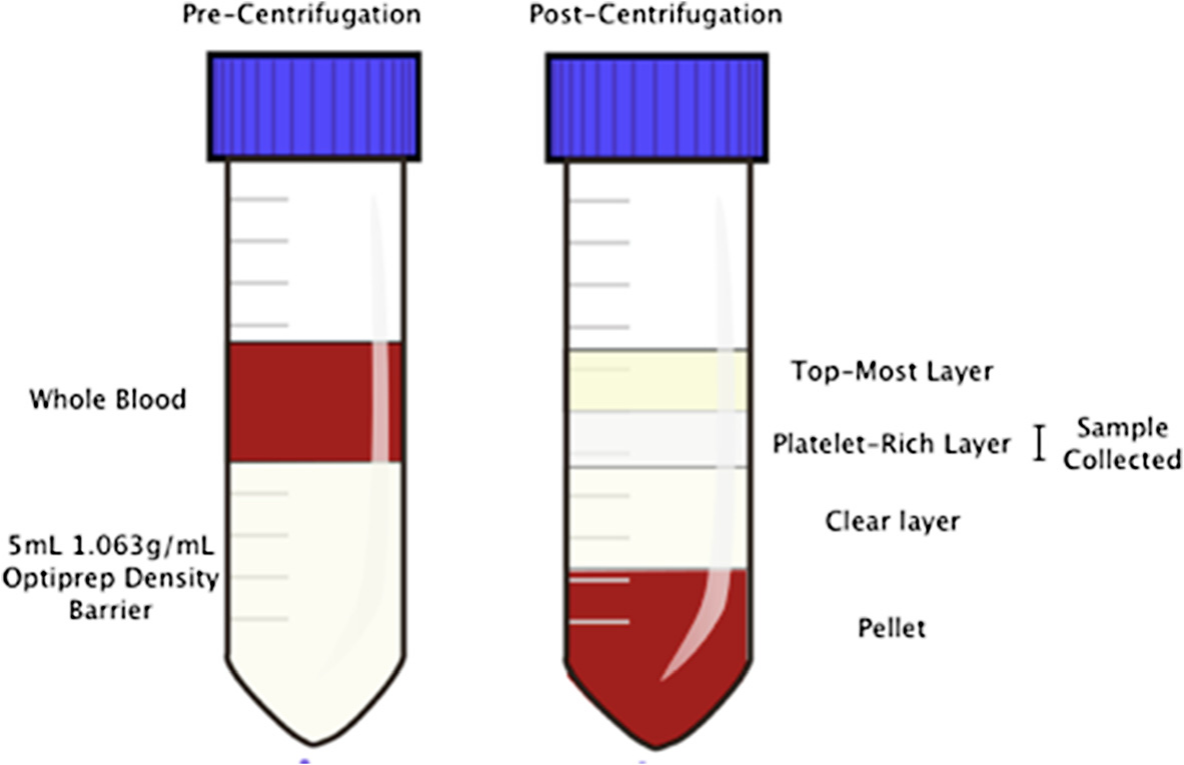
Density barrier layering before and after centrifugation with whole blood.

Following centrifugation of whole blood with a 1.063 g/mL density barrier, distinct layering was observed (Figure 1). After optimization of the OptiPrep density barrier centrifugation and platelet layer extraction steps, collection of 1 mL of the cloudy second layer consistently yielded a highly concentrated platelet fraction with an average purity of 99.99 ± 0.01% (mean ± standard deviation) by manual count, and average platelet recovery of 51.56 ± 9.66%. These platelet fractions were highly pure with only 100.07 ± 48.77 leukocytes per 10^8^ platelets. The original whole blood samples had cell counts, on average, of 2,457,405 ± 746,380 leukocytes per 10^8^ platelets. Therefore, on average, optimized density barrier centrifugation resulted in a 24,574-fold decrease of the leukocyte to platelet ratio from whole blood.

### Reduction of leukocyte contamination by bead separation

Throughout optimization of the OptiPrep density barrier purification technique, CD45-labeled Dynabeads were tested as a method to remove additional residual leukocytes. An average 95-fold decrease in leukocyte contamination was seen for samples containing high levels of contaminating cells. In samples containing an already low amount of contaminating leukocytes such as those seen with the OptiPrep density barrier centrifugation, there was no added benefit of continuing with the additional purification step using the leukocyte-specific beads due to additional platelet loss from the bead separation process.

### Purity, contamination, and assessment of activation by flow cytometry

**Figure 2:**
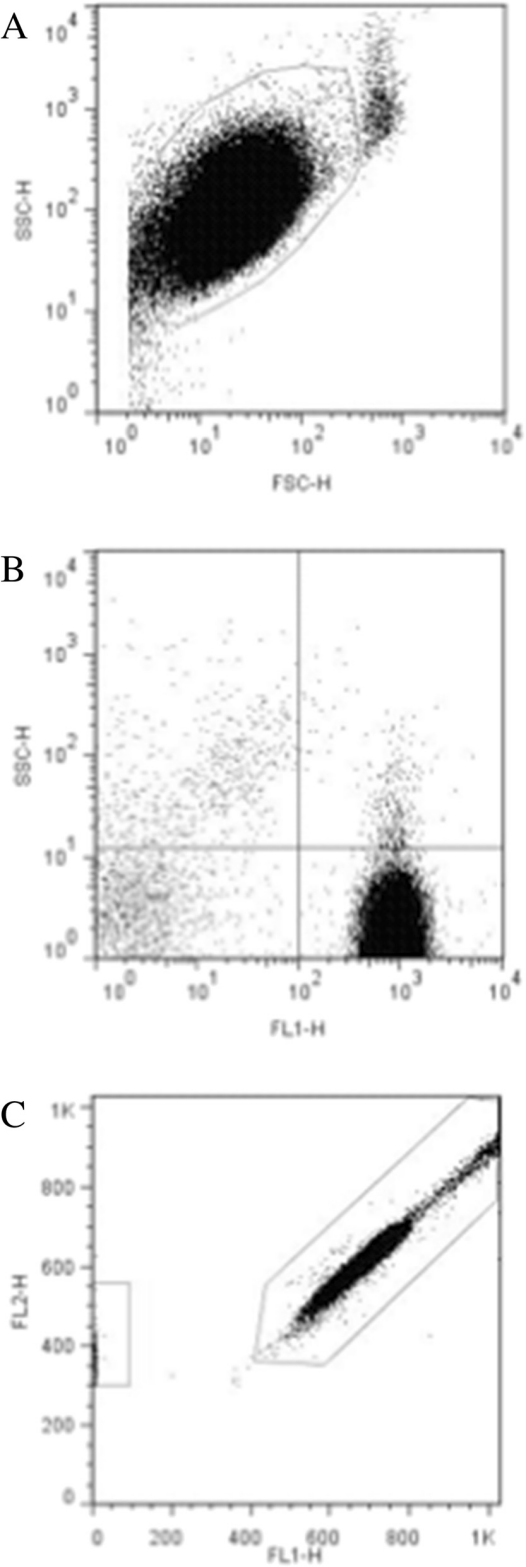
**Flow cytometric analyses of optimized platelet samples after density barrier centrifugation. (A)** Log forward and side scatter chart of platelet population (gated) with minimal contaminating cells. **(B)** Fluorescence chart of CD45 (FL2) and CD61 (FL1) antibody-labeled samples. Average CD61^+^: 99.47 ± 0.21% (n = 3). Average CD45^+^: 0.19 ± 0.04% (n = 3). **(C)** LeucoCOUNT chart for sample showing residual leukocytes (left gate) and LeucoCOUNT beads (right gate).

**Figure 3:**
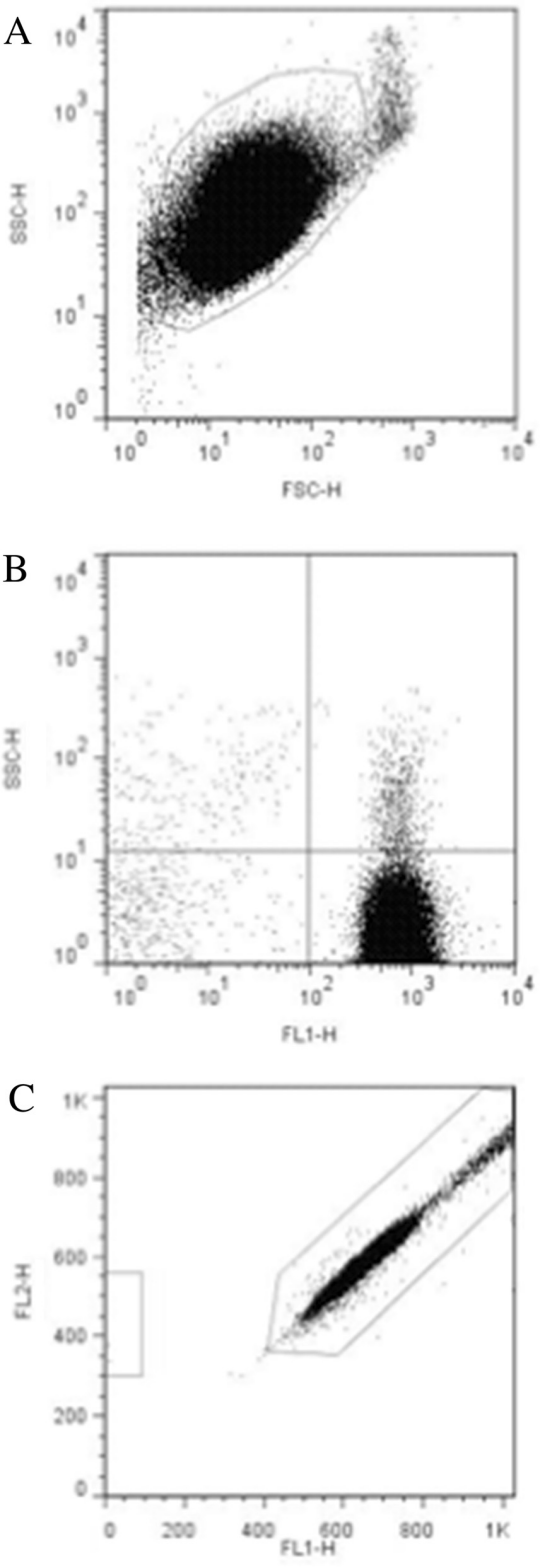
**Flow cytometric analyses of optimized platelet samples after CD45**-**labeled Dynabead incubation. (A)** Log forward and side scatter chart of platelet population (gated) of platelet sample with minimal contaminating cells. **(B)** Fluorescence chart of CD45 (FL2) and CD61 (FL1) antibody-labeled sample. Average CD61^+^: 97.99 ± 1.38% (n = 12). Average CD45^+^: 0.43 ± 0.33% (n = 12). **(C)** LeucoCOUNT chart for sample after density barrier centrifugation showing residual leukocytes (left gate) and LeucoCOUNT beads (right gate).

**Figure 4:**
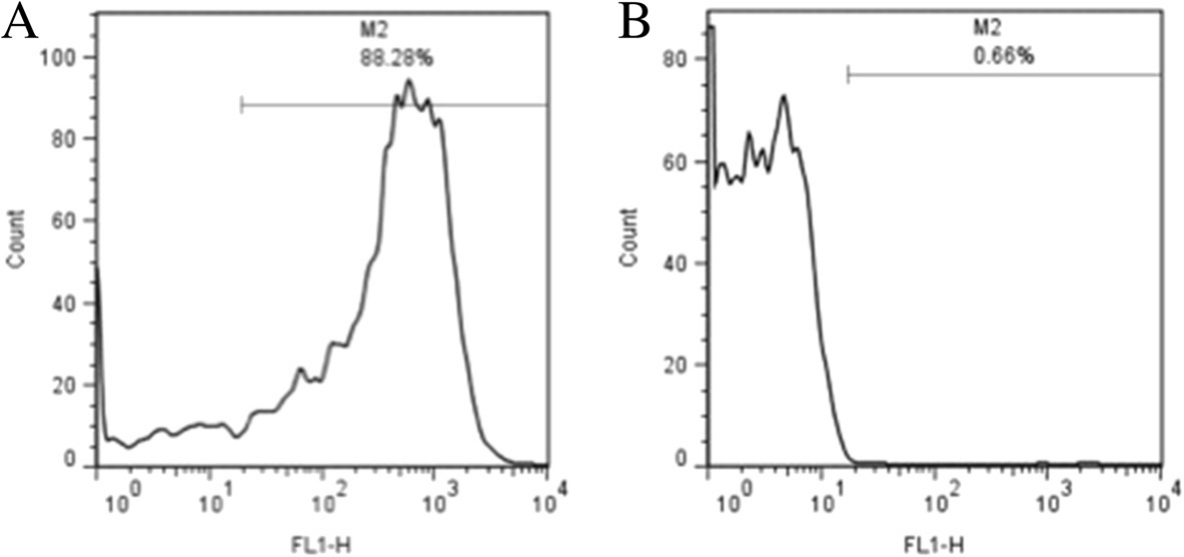
**Flow cytometric analyses of platelet activation by Annexin V labeling. (A)** Collagen activated platelets. **(B)** Platelet sample after density barrier centrifugation.

**Figure 5:**
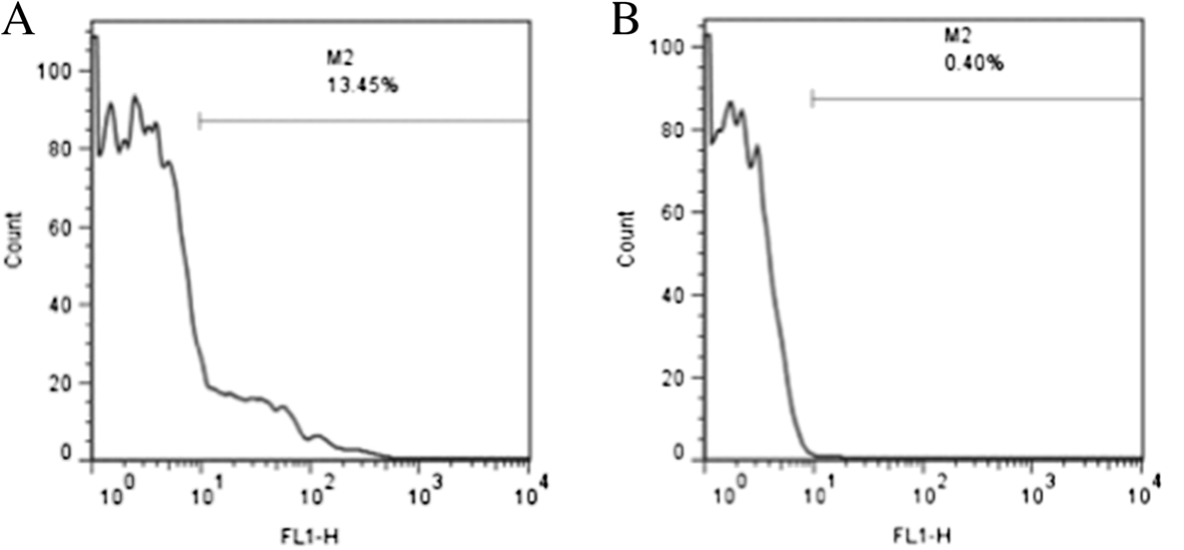
**Flow cytometric analyses of platelet activation by P**-**selecting labelling. (A)** Thrombin activated platelets. **(B)** Platelet sample after density barrier centrifugation.

Flow cytometric analysis of the platelet samples obtained after OptiPrep density barrier centrifugation indicated, on average, 99.47 ± 0.21% CD61-positive events and 0.19 ± 0.04% CD45-positive events. After incubation with CD45-labeled Dynabeads the samples had, on average, 98.84 ± 0.03% CD61-positive events and 0.27 ± 0.03% CD45-positive events (Figures 2 and 3). Platelet sample activation was evaluated using Annexin V and P-selectin labeling by flow cytometry (Figures 4 and 5). After initial centrifugation with the 1.063 g/mL density barrier, the platelet sample had 0.66% annexin positivity and 0.4% P-selectin positivity, and after incubation with CD45-labeled beads there was 3.00% Annexin positivity. Because of the platelet function inhibitory effect of the PECT anticoagulant, whole blood was collected in citrate to acquire PRP for use as positive controls by activating with thrombin and collagen. Thrombin activated platelets had 25.18% Annexin positivity and 13.44% P-selectin positivity while platelets activated by collagen had 88.28% Annexin positivity and 0.66% P-selectin positivity.

## Discussion

The described technique allows the purification of canine platelets using density barrier centrifugation and yields a highly pure platelet sample with very low leukocyte contamination (99.99 ± 0.01% platelet purity with only 100 leukocytes per 10^8^ platelets). This technique leaves platelets in a resting, non-activated state as assessed with Annexin V and P-selectin labeling by flow cytometry. Minimal centrifugation steps and sample handling is involved along with no additional need for filtering or special aphaeresis equipment. Moreover, this procedure is quick and efficient, resulting in a platelet recovery of approximately 52%, and can be performed on small, clinically practical whole blood sample volumes (3–5 mL).

Cell counting should be performed following the density barrier centrifugation step, and if residual leukocytes are seen in the counting chamber, it is recommended that the sample be incubated with CD45-labeled Dynabeads for removal of residual leukocytes to increase the purity of the platelet sample. Our results show that with this procedure residual leukocytes can be further reduced 95-fold (up to 586-fold maximum observed with optimized protocol) with minimal platelet loss.

During our optimization trials, we determined that the volumes and the ratio of whole blood to OptiPrep density barrier should be carefully maintained. Adding more or less than 3 mL of blood on top of the 5 mL of density barrier can cause poor layer separation, resulting in lower platelet purity and recovery. It is also vitally important to handle samples with care as to avoid any disturbance or mixing of the post-centrifugation layers, which may re-contaminate the platelet layer.

Little comparable data exists in the current literature in regards to veterinary species, including the dog. In humans however, density gradient centrifugation methods for platelet purification are well established, but are aided by aphaeresis-derived platelet concentrates obtained from large whole blood volumes [2,11,12]. One example of human platelet purification is the 1990 study from Ford et al. which used a 1.063 g/mL OptiPrep density barrier centrifugation of human whole blood to achieve a platelet recovery of 70% with 125 leukocytes per 10^8^ platelets [12]. More recently, Birschmann et al. achieved platelet samples with 21.9 and 1.5 leukocytes per 10^8^ platelets using pre-purified PRP and apheresis-derived platelet concentrates over layered OptiPrep density barriers, respectively [2]. In clinical veterinary settings, the collection of large blood volumes from single clinical patients is neither safe nor ethical and aphaeresis equipment is neither easily accessible nor cost effective. Here we describe a platelet purification technique that uses low clinically relevant volumes of canine whole blood and yields ultra-pure non-activated platelets with minimal leukocyte contamination.

## Conclusion

This study details an efficient technique for purification of canine blood platelets that results in ultra-pure resting platelet samples derived from small, clinically reasonable whole blood volumes. These ultra-pure resting platelet samples are thus suitable for use in multiple downstream analyses including proteomic and transcriptomic methodologies for the study of platelet physiology and functional roles in naturally occurring diseases in dogs.

## Methods

### Platelet purification

Healthy female intact Walker hound dogs were used in this study. The dogs were not exposed to any medications or vaccines for at least two months before initiation of the study. Animal use was approved by the Mississippi State University Institutional Animal Care and Use Committee and was in compliance with the requirements at a facility accredited by the American Association for Accreditation of Laboratory Animal Care. Whole blood samples were collected by jugular venipuncture with a 20-gauge needle directly into a glass vacutainer tube containing 0.5 mL of PECT medium (94 nM prostaglandin E_1_, 0.63 mM Na_2_CO_3_, 90 mM EDTA, and 10 mM the ophylline) that had an approximate draw of 5 mL. Sample preparation was initiated within one hour on the day of collection and initial blood cell counts were performed by an automated cell counter (Cell-Dyn 3700, Abbott Laboratories, Abbott Park, IL, USA).

A 1.063 g/mL density barrier was created by combining 5 mL of 1.320 g/mL 60% iodixanol stock solution (OptiPrep density gradient medium, Sigma-Aldrich, Saint Louis, MO, USA) with 22 mL diluent (0.85% NaCl, 20 mM HEPES-NaOH, pH 7.4, 1 mM EDTA). For platelet separation, 3 mL of each sample were layered over 5 mL of the 1.063 g/mL density barrier. Layered samples were then centrifuged at 350 *g* for 15 minutes at 20°C in a swinging bucket rotor with the brake turned off [11,12].One milliliter of the concentrated, opaque platelet fraction was collected as illustrated (Figure 1). Further removal of residual contaminating leukocytes was performed by coupling magnetic beads (Sheep anti-rat IgG Dyna beads, Invitrogen Dynal, Oslo, Norway) with rat anti-dog CD45 antibody (AbD Serotec, Raleigh, NC, USA), incubating the beads with the platelet sample for thirty minutes at room temperature with gentle rotation, and placing the mixture in a specialized magnet according to the manufacturer’s protocol. Cells in before and after bead depletion samples (platelets, leukocytes, and erythrocytes) were counted manually using a hemocytometer (improved Neubauer, Hausser Scientific Co., Harsham, PA, USA) to assess sample recovery and purity.

### Flow cytometry

Flowcyto metric analysis was performed with a flowcyto meter using Cell Quest Pro software (FACScalibur, BD Biosciences, San Jose, CA, USA). In order to assess sample purity, double staining with RPE-anti-CD45 (Rat anti-dog CD45: RPE, MCA1042PE, AbD Serotec, Raleigh, NC, USA) and FITC-anti-CD61 (Mouse anti-human CD61: FITC, BD Pharmingen, San Jose, CA, USA) were performed. One hundred micro liters of sample were incubated with 10 μL of anti-CD45 and 20 μL of anti-CD61 for 20 minutes at room temperature. The samples were washed and resuspended in phosphate buffered saline for analysis. Samples were displayed on log forward-angle versus log side-angle light scatter plots. Gates were determined to include all blood cells and results visualized on a dual color quadrant.

We also employed an automated leukocyte count technique, which relies on propidium iodide DNA staining and on test tubes containing precise numbers of fluorescent beads to quantify the residual leukocyte, according to manufacturer’s protocol (Leuco COUNT Reagent Kit, BD Biosciences). For each sample 30,000 bead events were counted.

Platelet activation was assessed using fluorescent-labeled Annexin V (FITC Annexin V, BD Biosciences) and RPE-anti-CD62P (P-selectin) (Mouse anti-human CD62P: RPE, MCA2419, AbD Serotec) binding and flow cytometric analysis. Annexin V binds exposed phosphatidylserine on the platelet membrane, a platelet membrane phospholipid that is trans located from the inner membrane leaflet to the exposed outer surface during platelet activation [12]. P-selectin is a platelet granule membrane protein that translocates to the outer membrane surface after activation and granule release [13]. For Annexin V labeling, around 5 million platelets (after OptiPrep and after Dynabeads) were spun down and resuspended in binding buffer before staining with 1:10 diluted Annexin V according to the manufacturer’s protocol. For P-selectin labeling, 20 uL of RPE-anti-CD62P antibody were added to platelet samples the same as described above for CD45 and CD61 labeling. For positive controls, platelets were activated with 1 U/ml of thrombin and 10 ug/mL collagen. Platelet populations were displayed on log forward-angle versus log side-angle light scatter plots. Gates were adjusted to platelet populations, and 5,000-gated events were recorded for each labeling. Expression was quantified by the percent of positive events for Annexin V and P-selectin.

## Competing interests

The authors declare that they have no competing interests.

## Authors’ contributions

SAT performed the experiments, interpreted the data, and wrote the manuscript. SCB performed the flow Cytometry and assisted with project coordination and manuscript preparation. JT facilitated sample collection. CB and KVL conceived the study. CB provided expert knowledge and assisted data interpretation. JT, KVL, and CB helped to draft the manuscript. CB is the project coordinator and the major professor of SAT. Study was part of SAT graduate work. All authors read and approved the final manuscript.
